# Rich Repertoire of Quorum Sensing Protein Coding Sequences in CPR and DPANN Associated with Interspecies and Interkingdom Communication

**DOI:** 10.1128/mSystems.00414-20

**Published:** 2020-10-13

**Authors:** Charles Bernard, Romain Lannes, Yanyan Li, Éric Bapteste, Philippe Lopez

**Affiliations:** a Institut de Systématique, Evolution, Biodiversité (ISYEB), Sorbonne Université, CNRS, Museum National d’Histoire Naturelle, Paris, France; b Unité Molécules de Communication et Adaptation des Micro-organismes (MCAM), CNRS, Museum National d’Histoire Naturelle, Paris, France; Dalhousie University

**Keywords:** CPR, DPANN, interkingdom signaling, microbial communication, microbiology, quorum sensing

## Abstract

The bacterial candidate phyla radiation (CPR) and the archaeal DPANN superphylum are two novel lineages that have substantially expanded the tree of life due to their large phylogenetic diversity. Because of their ultrasmall size, reduced genome, and lack of core biosynthetic capabilities, most CPR and DPANN members are predicted to be sustained through their interactions with other species. How the few characterized CPR and DPANN symbionts achieve these critical interactions is, however, poorly understood. Here, we conducted an *in silico* analysis on 2,597 CPR/DPANN genomes to test whether these ultrasmall microorganisms might encode homologs of reference proteins involved in the synthesis and/or the detection of 26 different types of communication molecules (quorum sensing [QS] signals), since QS signals are well-known mediators of intra- and interorganismic relationships. We report the discovery of 5,693 variants of QS proteins distributed across 63 CPR and 6 DPANN phyla and associated with 14 distinct types of communication molecules, most of which were characterized as interspecies QS signals.

**IMPORTANCE** The selection of predicted genes for interspecies communication within the CPR and DPANN genomes sheds some light onto the underlying mechanisms supporting their inferred symbiotic lifestyle. Also, considering the lack of core pathways such as the *de novo* synthesis of nucleotides or amino acids in the CPR and DPANN lineages, the persistence of these genes highlights how determinant social traits can be for the survival of some microorganisms. Finally, the considerable number of variants of QS proteins identified among the 69 CPR and DPANN phyla substantially expands our knowledge of prokaryotic communication across the tree of life and suggests that the multiplicity of “dialects” in the microbial world is probably larger than previously appreciated.

## Abstract (precis)

The selection of predicted genes for interspecies communication within the CPR and DPANN genomes sheds some light onto the underlying mechanisms supporting their inferred symbiotic lifestyle. Also, considering the lack of core pathways such as the *de novo* synthesis of nucleotides or amino acids in the CPR and DPANN lineages, the persistence of these genes highlights how determinant social traits can be for the survival of some microorganisms. Finally, the considerable number of variants of QS proteins identified among the 69 CPR and DPANN phyla substantially expands our knowledge of prokaryotic communication across the tree of life and suggests that the multiplicity of “dialects” in the microbial world is probably larger than previously appreciated.

## INTRODUCTION

The recent efforts at sequencing the DNA extracted from diverse environments enabled access to genomes of microorganisms with no isolated representatives, which together contributed to expand our vision of life’s diversity (1). Most of this expansion is attributable to the discovery of two novel microbial lineages, the candidate phyla radiation (CPR), estimated to account for more than 26% of the currently known bacterial diversity (2), and the archaeal DPANN superphylum (for Diapherotrites, Parvarchaeota, Aenigmarchaeota, Nanoarchaeota, and Nanohaloarchaeota) (3). Although little is known about these lineages, they already challenge our perspectives on the biology of prokaryotes; CPR and DPANN microorganisms have small to ultrasmall cell sizes (some can pass through 0.22-μm-pore-size filters [4]) and reduced genome sizes, and most of them lack core genes in pathways considered essential in other prokaryotic lineages, such as the *de novo* synthesis of nucleotides, amino acids or lipids (3, 5). These unusual traits suggest that the majority of these ultrasmall species might depend on other organisms to survive (6) or might even be obligate symbionts (5), a suggestion supported by the few endobiotic (7) and epibiotic (8, 9) relationships uncovered between a CPR or a DPANN and another microorganism(s). Nevertheless, the biological mechanisms underlying these interactions are currently poorly understood.

However, two recent studies suggest that interspecies communication via quorum sensing (QS) signals could be a new avenue of investigation to study how CPR and DPANN members may promote and/or maintain their interactions with other species (10, 11). The first study shows that the CPR member “*Candidatus* Saccharibacteria” TM7x HMT-952 interacts with and modulates the QS circuit of its obligate epibiont, Actinomyces odontolyticus XH001, which upregulates the biofilm formation pathway in this host and results into an increased stability of the TM7x-XH001 collective within dual-species biofilms (10). The second study hints at the presence of putative QS receptors in two DPANN genomes, “*Candidatus* Woesarchaeota” and “*Candidatus* Micrarchaeota” (11).

A canonical quorum sensing mechanism involves the collective emission of a diffusible signal molecule whose concentration correlates with the cellular density of the emitting microbial population. Upon reaching a threshold concentration (reflecting the quorum of the emitting population), the signal robustly binds to its cognate receptors, within or at the surface of individual cells, and is then transduced to coordinate the emergence of a collective behavior (1–14). Many symbionts, parasites, and even viruses rely on complete QS systems (one or several QS synthase[s] plus a cognate QS receptor) to orchestrate collective behaviors upon reaching a significant population density that either benefits to the host (15–17) or facilitates host invasion (18–20). Hence, QS often dynamically regulates the nature of the interaction between coevolving species. Importantly, the specificity, or secrecy, of a QS signal may range from the intraspecies to the interspecies and even interkingdom levels of recognition and may thus entail relationships of coinfluence within a community of organisms (21–23). It is also worthy of mention that microbiological entities can rely on QS receptors, either uncoupled or coupled with QS synthases, to eavesdrop on exogenous signals, which provides a means for collecting cues about the density or the physiological status of a host and to trigger biological processes accordingly (24–27). Conversely, orphan QS synthases may as well be selected for the influence that the produced signal might exert on the biology of other organisms, to the benefit of the emitting population. Indeed, some QS signals have been reported to inhibit or activate various receptors in nonemitting species and, therefore, to promote different types of host manipulation (28, 29).

Hence, considering that QS signals are key mediators of intra- to interorganismic communication modalities and that the survival of most CPR and DPANN members seems conditioned by their ability to promote and maintain interactions with other species, we designed a computational study to test whether CPR and DPANN might rely on QS genes to achieve their crucial interspecies interactions, as hinted by preliminary studies. In all CPR and DPANN genomes available on the NCBI website, we tested for the presence of homologs of experimentally characterized QS synthases and QS receptors, associated with 26 different types of QS signal molecules, to assess whether and via which “language(s)” CPR and DPANN do communicate.

## RESULTS

### CPRs and DPANNs encode homologs of reference proteins involved in diverse bacterial communication systems.

**FIG 1:**
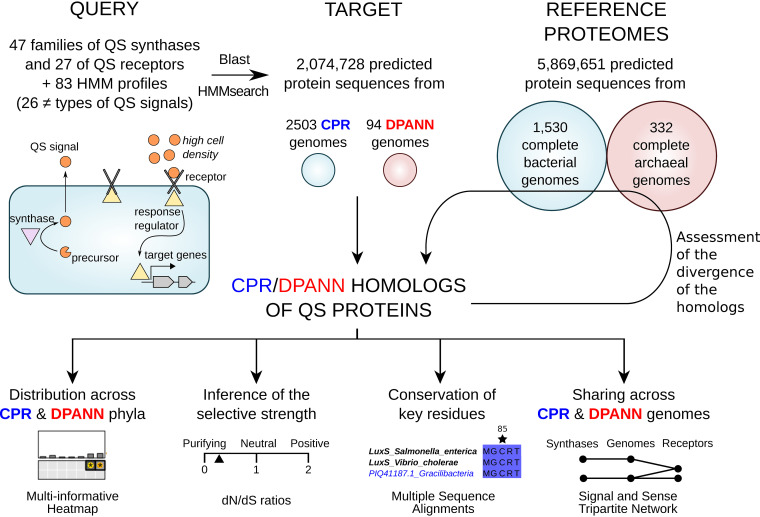
Study design. The first step consists in the identification of variants of QS synthases and receptors in the CPR and DPANN genomes available on the NCBI, using both BLAST queries and hidden Markov models (HMM) corresponding to reference, experimentally characterized QS proteins. Then, using BLAST, these CPR/DPANN homologs are queried against a library of protein sequences corresponding to the complete genomes of *Archaea* and representative *Bacteria* available on the NCBI, in order to assess how divergent their sequences are from their best match among well-studied prokaryotes. Further analyses are undertaken to (i) display the distribution of these homologs of QS proteins across CPR and DPANN phyla, (ii) estimate the selective strength acting on their genes, (iii) visualize the conservation level of key residues in their sequences whenever structural analyses have been conducted on the query QS proteins, and (iv) depict which CPR and DPANN genomes encode highly similar QS synthases (cosignalers) and receptors (cosensors).

A total of 423 reference protein sequences, distributed across 74 protein families (47 QS families of synthases and 27 families of receptors) and associated with 26 different types of QS signals (22 bacterial, 1 archaeal, 2 eukaryotic, and 1 viral), were used as a BLAST query data set (see Table S1 in the supplemental material). The 2,074,728 protein sequences predicted from the detected coding DNA sequences (CDS) of 2,503 CPR genomes and 94 DPANN genomes constituted the target data set. A homolog was defined as a protein whose sequence identity to a reference QS protein was no less than 25%, with over 75% mutual coverage and an E value below 1e−5 in a BLAST search (30, 31; see also Materials and Methods). These thresholds offer a good trade-off between functional reliability and permissive stringency, according to their application on a set of 76 experimentally validated LuxI synthases retrieved from the Sigmol Database (32) (see Fig. S1 in the supplemental material). A stringent “significance” label was assigned to homologs with an E value of <1e−20. Additionally, the homologs found by BLAST at a first iteration were allowed to serve as queries in a second BLAST search, as long as the remote homologs exhibited 75% mutual coverage with the initial query reference QS protein(s). When available, hidden Markov models (HMM) built from our own multiple-sequence alignments (MSAs) or retrieved from reference databases (Pfam and CATH_Gene3d) were used to check whether the CPR and DPANN sequences harbored the key, conserved residues of the query protein families (Fig. 1).

10.1128/mSystems.00414-20.1 FIG S1 Heat map of the mutual identities of 76 experimentally validated acyl/aryl-homoserine-lactone synthases retrieved from the Sigmol database. Download FIG S1, PDF file, 0.7 MB. Copyright © 2020 Bernard et al. 2020 Bernard et al. This content is distributed under the terms of the Creative Commons Attribution 4.0 International license.

10.1128/mSystems.00414-20.4 TABLE S1 Database of reference QS proteins and HMMs. Download Table S1, XLSX file, 0.04 MB. Copyright © 2020 Bernard et al. 2020 Bernard et al. This content is distributed under the terms of the Creative Commons Attribution 4.0 International license.

**FIG 2:**
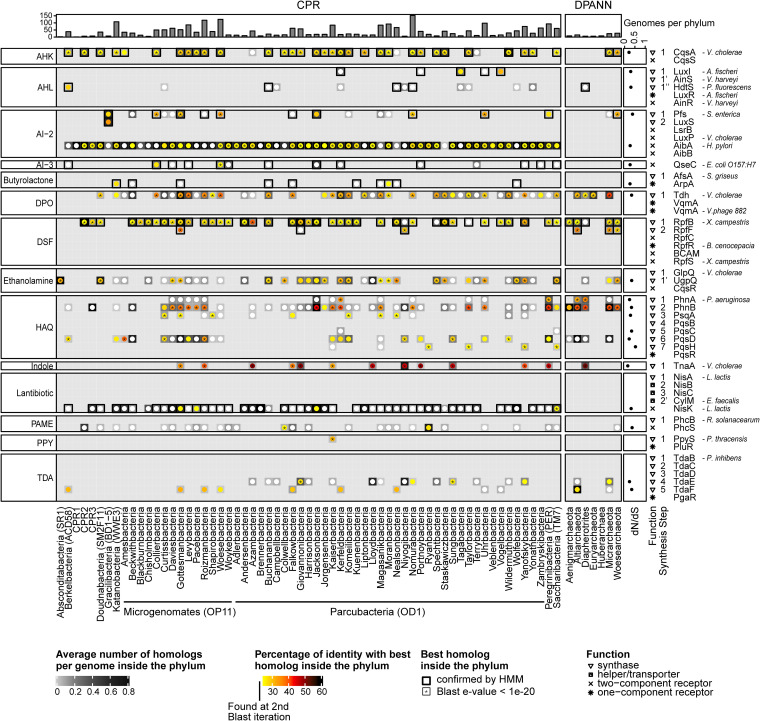
Multi-informative heat map of the QS synthases and receptors detected by BLAST in the CPR and DPANN phyla. Each column represents a phylum, and the histogram at the top displays the number of genomes per phylum. Rows represent query (reference) QS protein families, and the families are grouped by type of QS signals that they either produce or sense (label on the left). The species origin of the representative sequence of each reference protein family is given on the right of each row. The symbol adjacent to the name of each reference protein family indicates whether it corresponds to a QS synthase or a QS receptor (either one- or two-component system). Additionally, a special “synthesis step” label allows distinguishing families of reference synthases that are part of the same biosynthetic route from distinct families of synthases that might nonetheless catalyze the same biosynthetic step. The background in grayscale at each intersection of the heat map indicates the number of homologs of a reference protein family detected in a CPR or DPANN phylum, normalized by the number of genomes in the phylum. The color circle in the foreground displays the percentage of sequence identity between a query protein family and the best homolog detected in a CPR/DPANN phylum. An asterisk (*) indicates that this best homolog gave rise to a BLAST E value of <1e−20, a white circle indicates that it was found at a second BLAST iteration, and a bold rectangle indicates that it was detected by available HMM profiles as well. Finally, the plot on the right panel of the heat map displays the *dN*/*dS* ratio of each family of homologs.

We detected a total of 5,693 homologs, matching 24 query reference QS protein families and associated with 14 distinct QS signals; 2,003 proteins were found at the first BLAST iteration (24 families/14 signals), including 1,508 hits characterized by an E value of <1e−20 (18 families/12 signals), whereas 1,568 proteins identified by BLAST were also confirmed by available HMMs (10 families/8 signals) (Fig. 2; see also Tables S2 and S3). A total of 13 of the 14 detected QS signals were signals initially discovered in the *Proteobacteria* phylum, consistently with the wide spectrum of specificities reported for proteobacterial QS metabolites, ranging from intraspecies to interkingdom levels and suggesting that these signals might be produced or recognized by the phylogenetically distant CPR and DPANN lineages. The 14th and last type of QS signal identified in our data set corresponds to the γ-butyrolactones produced by *Actinobacteria*.

10.1128/mSystems.00414-20.5 TABLE S2 CPR/DPANN homologs of reference QS proteins identified by BLAST. Download Table S2, XLSX file, 0.7 MB. Copyright © 2020 Bernard et al. 2020 Bernard et al. This content is distributed under the terms of the Creative Commons Attribution 4.0 International license.

10.1128/mSystems.00414-20.6 TABLE S3 CPR/DPANN proteins identified by HMMs. Download Table S3, XLSX file, 1.6 MB. Copyright © 2020 Bernard et al. 2020 Bernard et al. This content is distributed under the terms of the Creative Commons Attribution 4.0 International license.

Overall, the 24 families of CPR/DPANN homologs of reference QS proteins are distributed across 63 CPR and 6 DPANN phyla; 19 families correspond to homologs of proteins involved in the biosynthesis of QS signals whereas 5 other families correspond to homologs of reference QS receptors (Fig. 2). These results suggest that CPR and DPANN members might emit and sense communication molecules, presumably to influence and monitor the biology of their hosts and neighbors.

### The vast majority of the sequences of the CPR and DPANN variants of QS proteins are divergent and under strong selective pressure.

In order to better characterize the CPR/DPANN homologs of reference QS proteins, we launched a BLAST search against the protein sequences encoded by the 332 complete genomes of *Archaea* and the 1,530 representative genomes of *Bacteria* available at the NCBI database to identify their best match in reference prokaryotes (Materials and Methods; see also Table S2). For each CPR/DPANN homolog found at a first BLAST iteration, its sequence identities to its query reference QS protein and to its best match within reference prokaryotic proteomes were plotted against each other in a scatterplot (Fig. S2). It appears that the overwhelming majority of the CPR/DPANN homologs of reference QS proteins exhibit no more than 60% identity to any protein predicted from the complete genomes of 1,862 prokaryotes, thereby highlighting how divergent their sequences are from those of well-studied organisms. On another note, comparisons of the functional annotations between each reference QS protein, the best corresponding CPR/DPANN homolog, and its closest match in the reference prokaryotic proteomes did not reveal functional inconsistencies (Table S2). Together, these results reinforce the prediction that CPRs and DPANNs emit and sense communication molecules but also suggest that these molecules may differ slightly from well-characterized QS signals.

10.1128/mSystems.00414-20.2 FIG S2 Scatterplot of the sequence identities of each CPR/DPANN homolog with its corresponding reference QS protein (*y* axis) and its closest homolog identified in the proteomes of reference prokaryotes (*x* axis). Download FIG S2, PDF file, 2.3 MB. Copyright © 2020 Bernard et al. 2020 Bernard et al. This content is distributed under the terms of the Creative Commons Attribution 4.0 International license.

Importantly, although the CPR/DPANN QS-related homologs are divergent, we found that selection was inferred to act against changes in their protein sequence. Indeed, the *dN*/*dS* ratio (a metric relying on the ratio of the number of nonsynonymous mutations to the number of synonymous mutations along distinct coding sequences) corresponding to each family of homologs was always closer to 0 than to 1 (median = 0.3), which is indicative of strong, purifying selection acting on these genes (Materials and Methods) (the distribution of *dN*/*dS* ratios is shown in Fig. 2). This finding highlights the importance of the functions that the CPR/DPANN’s homologs of QS proteins actually support.

### CPR and DPANN are predicted to produce interspecies QS signals that might influence their hosts/neighbors.

Having uncovered the presence of 19 families of QS synthases in the CPR and DPANN lineages, we sought to characterize which types of communication molecules these proteins might contribute to produce. We found that these 19 families of QS synthases are distributed in the biosynthetic pathways of 10 distinct types of QS signals. For 2 of these 10 types of QS signals, namely, HAQ (hydroxy-alkyl quinolines) and TDA (tropodithietic acid), no complete biosynthetic pathways were identified in CPR/DPANN genomes (Fig. 2). Conversely, the biosynthetic routes for the 8 other types of QS signals are found complete in some CPR/DPANN genomes, in which they thus likely support the production of communication molecules.

Specifically, we identified that some CPRs and DPANNs might produce (i) AHK (alpha-hydroxyketones) via homologs of the CqsA, LqsA, and JqsA ketosynthases (33); (ii) AHL (acyl-homoserinelactones) via homologs of the LuxI (13) or the HdtS (34) families; (iii) pyrazines/pyrazinoles such as AI-3 (autoinducer-3) and DPO (3,5-dimethylpyrazin-2-ol) via homologs of the Tdh threonine dehydrogenase (35, 36); (iv) DSF (diffusible small factors) via the RpfB/RpfF pathway (37); (v) ethanolamine via homologs of the UgpQ glycerophosphoryl-diester-phosphodiesterase (38); and (vi) indole via homologs of the TnaA tryptophanase (39). In addition, some members of the CPR lineage might produce (vii) AI-2 (autoinducer-2) via the Pfs/LuxS pathway (22) and (viii) PPY (photopyrones) via homologs of the PpyS ketosynthase (40) (Fig. 2; see also Table 1).

**TABLE 1:** Proposed roles for the homologs of reference QS proteins found within the CPR and DPANN lineages

Signal	Reference protein family	No. of mapped phyla (BLAST)	No. of homologs (BLAST)	No. of homologs (HMM)	Conserved residue(s)	No. of remarkable CDS adjacencies	Possible roles for the CPR/DPANN homologs
AHK (alpha hydroxy- ketones)	CqsA synthase (Vibrio cholerae)	35 (CPR), 2 (DPANN)	362 (CPR), 11 (DPANN)	364 (CPR), 18 (DPANN)	Fig. 3A (42)	51 CqsA_homolog_/putative sensory kinase (with a CqsS sensory domain)	Switch persistence/escape from the host (as in Legionella pneumophila [28, 42]), modulation of eukaryotic motility (as in Vibrio cholerae and Legionella pneumophila [42–44]), inhibition of eukaryotic growth (as in *Janthinobacterium* [45]), …
AHL (acyl homoserine lactones)	LuxI synthase (Aliivibrio fischeri)	5 (CPR)	9 (CPR)	36 (CPR)	Fig. 3D (76)	18 LuxI_homolog_/putative sensory kinase	Persistence within the host (as in Brucella melitensis [46]), interspecies to interkingdom signaling (27), induction of multispecies biofilm formation (78), activation or inhibition of QS receptors in other prokaryotes, …
	HdtS synthase (*Pseudomonas fluorescens*)	12 (CPR), 1 (DPANN)	23 (CPR), 1 (DPANN)	83 (CPR), 3 (DPANN)	Fig. 3F (34)		
AI-2 (auto- inducer-2)	Pfs synthase (step 1) (*Saccharomyces enterica*)	16 (CPR), 1 (DPANN)	84 (CPR), 2 (DPANN)	56 (CPR), 1 (DPANN)		4 Pfs_homolog_/LuxS_homolog_	Signaling presence to other prokaryotes (22), facilitation of the incorporation into multispecies biofilm (41), …
	LuxS synthase (step 2) (*Saccharomyces enterica*)	1 (CPR)	4 (CPR)	6 (CPR)	Fig. 3C (77)		
	AibA receptor (Helicobacter pylori)	62 (CPR)	1,876 (CPR)	No reference HMMs			Estimation of the cellular density of the bacterial community (22), …
Pyrazines/pyrazinols	Tdh synthase (Vibrio cholerae)	41 (CPR), 4 (DPANN)	326 (CPR), 21 (DPANN)	No reference HMMs	Fig. 3B (74)		Interspecies (25) to interkingdom (36) signaling, …
DSF (diffusible small factors)	RpfB synthase (step 1) (*Xanthomonas campestris*)	40 (CPR), 5 (DPANN)	433 (CPR), 22 (DPANN)	619 (CPR), 34 (DPANN)		11 RpfB_homolog_/RpfF_homolog_	Interspecies to interkingdom signaling, regulation of motility, biofilm formation, iron uptake, virulence in other prokaryotes, elicitation of the innate immunity of plants, induction of the stringent response, and siderophore production in other bacteria (as in Burkholderia cenocepacia) (37), …
	RpfF synthase (step 2) (*Xanthomonas campestris*)	3 (CPR), 3 (DPANN)	6 (CPR), 7 (DPANN)	67 (CPR), 8 (DPANN)	Fig. 3E (77)		
Ethanolamine	UgpQ synthase (Vibrio cholerae)	37 (CPR), 7 (DPANN)	247 (CPR), 7 (DPANN)	No reference HMMs			Interkingdom signaling (38), …
Indole	TnaA synthase (Vibrio cholerae)	12 (CPR), 1 (DPANN)	61 (CPR), 2 (DPANN)	No reference HMMs			Inhibition of QS receptors in other prokaryotes (79), modulation of biofilm formation, motility and antibiotic resistance within microbial communities (47), …
PPY (photopyrones)	PpyS synthase (*Photorhabdus thracensis*)	1 (CPR)	3 (CPR)	No reference HMMs		3 PpyS_homolog_/putative sensory kinase	Cell clumping (as in Photorhabdus thracensis [40]), …
γ-Butyro- lactones	ArpA receptor (*Streptomyces griseus*)	10 (CPR)	17 (CPR)	63 (CPR)	Fig. 3G (49)		Eavesdropping on host QS molecules (53), …
Lantibiotics	NisK receptor (*Lactococcus lactis*)	51 (CPR)	454 (CPR)	71 (CPR), 2 (DPANN)			Efflux/resistance to exogenous lantibiotics (52), QS (50), …

**FIG 3:**
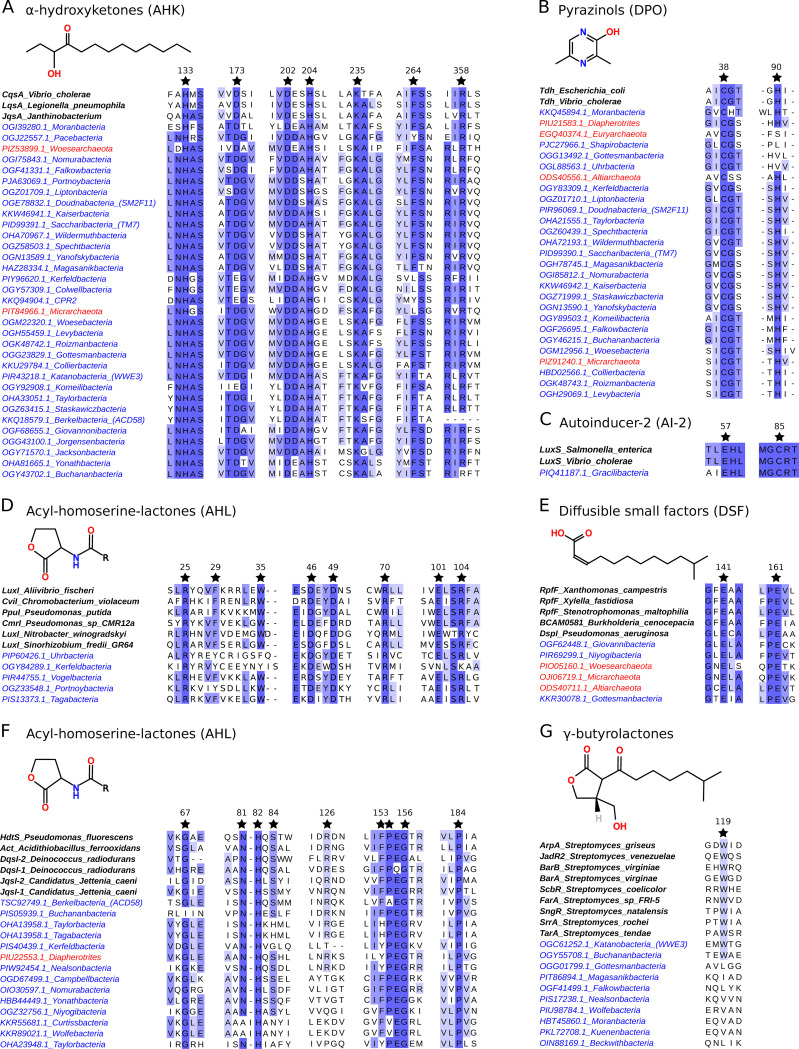
Partial multiple-sequence alignments of CPR/DPANN homologs with reference QS proteins to study the conservation of key residues. Wherever important residues (black stars) for the QS function have been identified in the sequences of reference QS proteins (names in bold), their level of conservation and the representative homologs of each CPR phylum (names in blue) and DPANN phylum (names in red) are displayed. (A) Ketosynthases (CqsA family): synthesis of α-hydroxyketones (structural study [42]). (B) Threonine dehydrogenases (Tdh family): synthesis of pyrazines and pyrazinols (structural study [74]). (C) *S*-Ribosylhomocysteine lyases (LuxS family): synthesis of AI-2 (structural study [75]). (D) AHL synthases (LuxI family): synthesis of acyl-homoserine lactones (structural study [76]). When not conserved, the aromatic F-29 and W-35 residues are substituted by other aromatic amino acids. (E) Enoyl-CoA-hydratases (RpfF family): synthesis of diffusible small factors (structural study [77]). (F) AHL synthases (HdtS family): synthesis of acyl-homoserine lactones (structural study [34]). (G) One-component receptors (ArpA family): sensing of γ-butyrolactones (structural study [49]).

For every case in which structural studies have been conducted on a reference QS protein family and led to the identification of residues that are essential for the biosynthesis of the QS signal(s), we assessed the level of conservation of these residues in the best homolog found within each CPR/DPANN phylum. Overall, homologs of CqsA, LuxI, HdtS, Tdh, LuxS, and RpfF were found to almost systematically exhibit the residues that are known to be important for the production of their respective type of QS signals (Fig. 3). When such was not the case, a key residue was often found to have been substituted by an amino acid of the same kind (e.g., the Trp-35 of the LuxI family was either conserved or substituted by another aromatic residue [Phe or Tyr]), hinting at alternative biosynthetic modalities in some CPR/DPANN’s homologs rather than at disrupted biosynthetic capabilities (Fig. 3).

We also paid attention to the synteny of the coding DNA sequences (CDS) of these homologs of QS synthases, because adjacent QS genes are likely to be functionally linked and to participate together in the QS function. We report that the *pfs* (*S*-adenosylhomocysteine nucleosidase) and *luxS* (*S*-ribosylhomocysteine lyase) genes encoding putative AI-2 synthases are found adjacent to each other in “*Candidatus* Gracilibacteria” from Crystal Geyser, UT. Likewise, the CDS of the homologs of the RpfB (fatty-acid-coenzyme A [CoA]-ligase) and RpfF (enoyl-CoA-hydratase) DSF synthases are found adjacent to each other in three genomes of “*Candidatus* Giovannonibacteria.” Furthermore, HMMs allowed us to identify 8 additional similar genomic contexts in “*Candidatus* Giovannonibacteria” and “*Candidatus* Harrisonbacteria” (Table S4). Of note, homologs of *rpfB* and *rpfF* are also present together, yet not adjacent, in 1 “*Candidatus* Gottesmanbacteria,” 1 “*Candidatus* Niyogibacteria,” 1 *“Candidatus* Altiarchaeota,*”* and 2 “*Candidatus* Woesearchaeota” genomes. Importantly, we also found that 51 CDS of the CqsA homologs in CPRs are in synteny with the CDS of proteins matching the HMM profile of the CqsS α-hydroxyketone receptor kinases, hinting at possible complete AHK-based QS systems in certain CPR genomes. Likewise, the CDS of all the homologs of PpyS as well as of 18 homologs of LuxI were found adjacent to a sensory kinase in some CPR genomes, thereby representing other candidates for complete QS systems (Table S4).

10.1128/mSystems.00414-20.7 TABLE S4 Variants of QS components adjacent to each other in CPR and DPANN genomes. Download Table S4, XLSX file, 0.05 MB. Copyright © 2020 Bernard et al. 2020 Bernard et al. This content is distributed under the terms of the Creative Commons Attribution 4.0 International license.

Finally, we looked at the literature to propose roles for these 8 types of communication molecules inferred to be secreted by CPRs and DPANNs. It appears that these families of molecules are known to mediate interspecies signaling (AI-2, the DPO pyrazinol, AHL, indole, DSF) and interkingdom signaling (AHK, AHL, the AI-3 pyrazine, DSF, ethanolamine) (Table 1). The secretion of these types of QS signals hence might help CPRs and DPANNs to promote and/or maintain their crucial interactions with other species. To give a few examples, the AI-2 signal emitted by some “*Candidatus* Gracilibacteria” CPR strains could facilitate their incorporation into multispecies biofilms, in agreement with the acknowledged association between the concentration of the AI-2 signal and the formation of mutualistic biofilms (41). Again, the AHK produced by CPRs and DPANNs might regulate the growth or the motility of eukaryotic hosts, to their own benefit (42–45). The possible AHK-based and AHL-based QS systems of certain CPR endobionts might control when to remain within or when to escape from the host according to the density of CPRs within the host’s cytoplasm (28, 42, 46). The biosynthesis of indole might also have been selected in some CPR and DPANN members for the various effects that this molecule exerts on microbial communities, such as modulation of biofilm formation, motility, virulence, antibiotic resistance, etc. (47) (Table 1).

### CPR and DPANN are predicted to detect exogenous communication molecules and to collect cues about their hosts/neighbors.

In addition to the 19 families of QS synthases, 5 families of CPR/DPANN homologs correspond to reference sensors of QS molecules (Fig. 2). Two of these five families, namely, homologs of QseC and PhcS, can potentially produce false positives for recognition of QS signal(s) because no homologs of QseC exhibit the characteristic glutamate- and aspartate-rich motifs responsible for AI-3 binding (48) and no homologs of PhcS match the N-terminal sensory region of PhcS that binds QS signals of methyl esters such as palmitic acid methyl ester (PAME) or myristic acid methyl ester (MAME). Conversely, the 3 other families, detected only in the CPR lineage, are likely to support recognition of QS signal(s).

Specifically, CPR members might sense (i) the γ-butyrolactones produced by potential actinobacterial hosts via homologs of ArpA (49); (ii) endogenous or exogenous peptide lantibiotics such as nisin, subtilin, and mersacidin via homologs of the NisK kinase (50); and (iii) the AI-2 QS molecule via homologs of the AibA QS receptor, recently identified in Helicobacter pylori (51) (Fig. 2; see also Table 1).

ArpA is a transcription factor with a DNA binding domain (C-terminal HTH motif) that becomes activated when its N-terminal sensory domain binds with γ-butyrolactone, notably via a key tryptophan residue at position 119 (49). All 17 homologs of ArpA harbor a canonical HTH domain, but the Trp119 residue is present only in the “*Candidatus* Katanobacteria” (WW3) and ”*Candidatus* Bachananbacteria” phyla (Fig. 3G). Hence, besides some members of the “*Candidatus* Katanobacteria” and “*Candidatus* Bachananbacteria” phyla which are highly likely to sense γ-butyrolactones, the other ArpA-encoding genomes of the CPR radiation might either bind with γ-butyrolactones despite the absence of Trp119 or bind with other types of communication molecules.

The examination of the genomic context of the CDS of the only homolog of NisK with an E value of <1e−20 (protein RYC73835.1 of a “*Candidatus* Saccharibacteria” strain) reveals that it forms a two-component system with an adjacent response regulator, flanked on both sides by tandem ABC transporters from the BceA-BceB family. This genomic context is the signature of a resistance/efflux system for exogenous bacteriocins (52).

To sum up, the CPR homologs of the ArpA family are predicted to support eavesdropping on host-produced, likely actinobacterial QS molecules, presumably as a means to collect cues about the density status of hosts and to trigger pathways accordingly. This prediction is consistent with the relation of epibiosis between a CPR member (the TM7x “*Candidatus* Saccharibacteria”) and an actinobacterium (Actinomyces odontolyticus) as previously reported (8). As a matter of fact, our HMM of the ArpA protein family actually matches a protein encoded by TM7 oral taxon 349 (protein TWP21250.1, E value = 2e−18, Table S3). Homologs of NisK might enable some CPRs to sense and pump out exogenous peptide lantibiotics, but they could as well be specific to endogenous lantibiotics and mediate intraspecies QS as in Lactococcus lactis or Bacillus subtilis (48). Last but not least, the 1,876 CPR homologs of AibA were detected by BLAST in all CPR phyla, except in the “*Candidatus* Absconditabacteria” phylum (Fig. 2; see also Table S2). Considering that the AI-2 QS signal is produced by bacteria from many different phyla, the functional validation of all the AibA homologs as AI-2 sensors would imply that almost all CPR members are able to estimate the cellular density of their bacterial community.

Finally, in addition to the homologs of ArpA, NisK, and AibA identified by BLAST, we found interesting the match of 28 CPR proteins (distributed across the “*Candidatus* Kerfeldbacteria” and “*Candidatus* Wolfebacteria” CPR phyla) with the HMM profile built from the multiple-sequence alignment of the PAS4 domain of 35 receptors of the VqmA family (DPO sensing) (Table S3). Indeed, PAS4 domains are assumed to bind with hormone-like molecules, notably from eukaryotic hosts, and therefore to be involved in both QS and host sensing (40).

### Signal and sense tripartite network.

**FIG 4:**
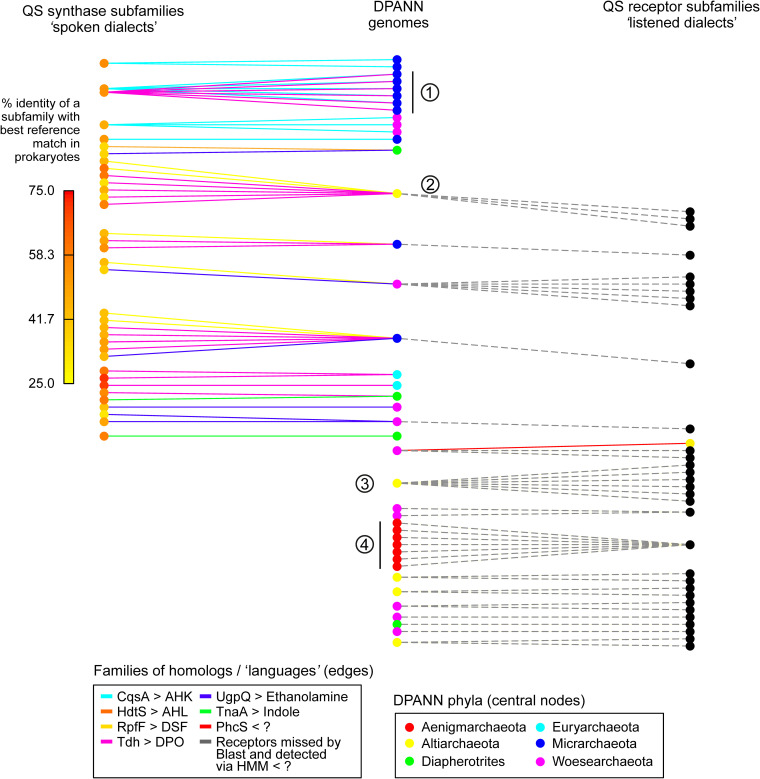
Signal and sense tripartite network in the DPANN lineage. This representation allows appreciating which and how many QS signals the different DPANN members might secrete and/or eavesdrop on at the genome level. The central layer of nodes corresponds to DPANN genomes. The left layer of nodes corresponds to subfamilies of homologs of QS synthases (family of homologs subpartitioned at 90% sequence identity), and the right layer of nodes to subfamilies of homologs of QS receptors. Hence, wherever two “Genome” nodes are connected to a left node, it means that they encode highly similar variants of QS synthases and are thus likely cosignalers. Likewise, wherever two “Genome” nodes are connected to a right node, it means that they are likely cosensors. The color of the edges allows identifying the initial family from which a subfamily has been further defined. Hence, left and right nodes could depict “spoken” and “listened” “dialects,” respectively, whereas the color of the edges could depict the “languages” from which these dialects derive. Of note, dashed edges correspond to putative QS receptors identified by HMM and missed by BLAST. The color of the central nodes indicates the phylum of each genome. The color of the subfamilies identified by BLAST indicates their respective average percentages of identity with the best matches in reference prokaryotes; yellow nodes thus highlight sequences that are more divergent than those highlighted by red nodes. Examples: (1) genomes predicted to “speak” the same “dialects” of the AHK and DPO languages; (2) genome predicted to “speak” several dialects of the DPO and DSF languages and to sense several signals; (3) genome predicted to “listen to” several dialects; (4) genomes predicted to “listen to” the same dialect. The tripartite network of the CPRs is displayed in Fig. S3.

To summarize the results of our analysis, we introduce “Signal and Sense Tripartite Networks” representing a novel framework that describes, for each genome (central nodes), which type of QS signal(s) it is predicted to produce (left nodes), and which type of QS signal(s) it is predicted to sense (right nodes). Specifically, we partitioned each relevant family of CPR/DPANN homologs of either QS synthases or QS receptors into subfamilies using a clustering threshold at 90% sequence identity (Table S5). Hence, whenever two nodes of the central layer “Genome” are connected to the same node of the left layer “Synthase,” it means that these two genomes are likely to produce the same QS molecule and thus to be cosignalers and to speak the same “dialect” of a broader “language,” each “language” corresponding to a family of QS molecules. Likewise, genomes connected to the same node of the right layer “Receptor” highlight likely cosensors. This depiction allows appreciating which and how many QS signals the different CPR and DPANN members might secrete and/or eavesdrop on at the genome level. The “Signal and Sense Tripartite Network” of the DPANN genomes is given in Fig. 4, whereas the network of the CPR genomes, much bigger, is given in Fig. S3.

10.1128/mSystems.00414-20.3 FIG S3 Signal and sense tripartite network in the CPR lineage. Download FIG S3, PDF file, 0.6 MB. Copyright © 2020 Bernard et al. 2020 Bernard et al. This content is distributed under the terms of the Creative Commons Attribution 4.0 International license.

10.1128/mSystems.00414-20.8 TABLE S5 Signal and sense tripartite network data. Download Table S5, XLSX file, 0.4 MB. Copyright © 2020 Bernard et al. 2020 Bernard et al. This content is distributed under the terms of the Creative Commons Attribution 4.0 International license.

## DISCUSSION

Our survey results show a rich repertoire of homologs of QS proteins encoded by 2,205 CPR and 66 DPANN genomes. We displayed the distribution of these proteins across the different CPR and DPANN phyla (Fig. 2) and found that they are associated with 14 different QS signals, of 25 tested. Since the CPR and DPANN lineages account for a substantial amount of phylogenetic diversity, the functional characterization of these QS proteins could significantly expand our knowledge of microbial communication across the tree of life. Specifically, QS signals have been characterized so far, to the best of our knowledge, in at least 10 microbial phyla, distributed across *Bacteria* (14, 22, 53, 54), *Archaea* (55, 56), *Eukarya* (57), and viruses (17), and our study on the CPR and DPANN lineages suggests that this spectrum could potentially expand to up to 69 additional prokaryotic phyla.

Interestingly, the majority of the sequences of the CPR/DPANN variants of reference QS proteins are divergent from those of proteins encoded by reference genomes (see Fig. S2 and Table S2 in the supplemental material). In this regard, heterologous expression of CPR/DPANN putative QS synthases in model prokaryotes would be particularly interesting because it could lead to the discovery of new communication molecules. Given that they may act as antagonists of known QS receptors in pathogens, this could lead to new anti-infective strategies.

From an evolutionary viewpoint, considering that CPR and DPANN genomes are suggested to have undergone genome reduction, the persistence of QS genes which would allow them to signal their presence, influence their neighbors, and collect social cues might underline the prime importance of the role played by social traits in their survival. Our predictions could hence convey the strong message that social traits can sometimes be more critical for the survival of certain species than many traits that are considered essential in most microbes and yet are absent in most CPR and DPANN members, such as, for example, the *de novo* synthesis of nucleotides, lipids, or amino acids.

Our results also pave the way to functional studies that could help decipher the underlying functions supporting the inferred symbiotic/parasitic lifestyles of CPR and DPANN members. Indeed, our survey results suggest that many partial or complete QS systems in CPR/DPANN genomes support interorganism and cross-kingdom communication (Table 1), which could be determinant in the promotion and/or the maintenance of the critical interactions of CPR and DPANN with other species. Specifically, depending on the different combinations of QS components (QS synthase[s] and/or QS receptor) identified in the CPR and DPANN genomes, we predict three types of communication modalities for these microorganisms: quorum sensing, presence signaling (or manipulation), and eavesdropping on exogenous signals.

Density-dependent mechanisms of QS are typically supported by complete QS systems (QS synthase[s] plus QS receptor). In this respect, we identified genes of QS synthases in CPR genomes that are adjacent to transmembrane kinases harboring a binding domain for the QS signal predicted to be synthesized (Table S4). This genomic context is typical of a complete QS system, since QS synthases and their cognate QS receptors are often colocalized in microbial genomes. This being said, the selection of a complete QS system of genes (synthase[s] plus receptor) to regulate biological processes in a cell density-dependent manner presupposes that the microbial population expressing the QS system occasionally encounters a high cellular density. Consistently, the prediction of complete QS systems in CPR genomes is reinforced by several studies reporting that some CPR species are found to be abundant under some conditions. For example, “*Candidatus* Sonnebornia yantaiensis” is usually found at low abundance in freshwater but is sometimes present by the thousands in the cytoplasm of paramecia (7). Again, it has been reported that the relative abundance of “*Candidatus* Saccharibacteria” is about ∼1% in healthy human oral cavities but can increase to up to 21% of the whole microbial community in cases of periodontal diseases (58, 59). Together, these findings suggest that the putative QS mechanisms of certain CPRs might be triggered when they reach a critical density within the cytoplasm of their hosts or within multispecies biofilms.

Apart from these predicted complete QS genetic systems, we also identified variants of QS synthases and QS receptors with no detected cognate QS component in some genomes. Although “not detected” does not necessarily imply “not present,” we further discuss different scenarios that could explain the selection of uncoupled QS synthases and uncoupled QS receptors. Orphan QS synthases could be selected for the advantageous influence that the produced QS signal(s) would exert on the behavior of other species, to the benefit of the emitting population. For instance, the QS signals that they produce could act as antagonists of QS receptors upregulating microbial defense mechanisms in other species. Also, since most of the homologs of QS synthases in the genomes of CPR and DPANN are associated with interspecies and interkingdom signaling, the QS signal(s) that they would produce could mediate a wide range of influences within microbial communities; α-hydroxyketones could modulate the motility of eukaryotic cells, AI-2 could coerce other cells to produce multispecies biofilms, indole could modulate many biological processes inside a community of microorganisms (motility, virulence, antibiotic resistance, etc.). The converse case, namely, the selection of orphan QS receptors in CPR genomes (notably for AI-2 and γ-butyrolactones), is not rare in nature (24, 25, 27) and is explained by the fact that these receptors can enable eavesdropping on the density status of hosts or neighbors.

A recent functional study provided insights into the way in which CPR could make use of such orphan receptors to regulate their interactions with other microorganisms (10). That study showed that Actinomyces odontolyticus XH001, the obligate epibiont of the “*Candidatus* Saccharibacteria” TM7x HMT-952 CPR, relies collectively on the emission (via the LuxS_homolog_ QS synthase) and perception (via the LsrB_homolog_ transmembrane QS receptor) of the AI-2 QS signal to orchestrate biofilm formation in a density-dependent manner. Those authors further report three fascinating results: (i) upon association with the TM7x CPR, the most highly induced gene in XH001 was the *lsrB* QS receptor; (ii) upon deletion of the *luxS* QS synthase in XH001, TM7x cells no longer induced significant upregulation of *lsrB;* (iii) wild-type XH001 cocultured with TM7x displayed significantly increased biofilm formation compared to XH001 alone and to XH001_ΔluxS_-TM7x and XH001_ΔlsrB_-TM7x cocultures. A parsimonious hypothesis to account for these observations could be that upon detection of the host-produced AI-2 QS signal, perhaps via a homolog of AibA (Fig. 2), TM7x cells would perceive when the XH001 population reached a sufficient cellular density compatible with interspecies biofilm formation. Thereupon, they would coerce XH001 cells, through *lsrB* upregulation, to be themselves more sensitive to the AI-2 signal, misleading these hosts to overestimate their quorum and to subsequently precipitate the induction of the biofilm formation pathway to the benefit of the stability of the TM7x-XH001 collective. This hypothesis is consistent with the previously reported observations that the AI-2 QS signal promotes interspecies interactions between periodontopathogens via enhanced expression of biofilm molecules (60) and that the relative abundance of “*Candidatus* Saccharibacteria” in the oral microbiome is about 1% in healthy human cavities but can increase to 21% in case of periodontal diseases (58, 59).

To conclude, our *in silico* analysis opens the possibility of exciting perspectives in CPR/DPANN biology and in prokaryotic QS research in the foreseeable future. In the long term, deciphering QS processes in the CPR and DPANN novel lineages will be an important milestone toward understanding how these microorganisms achieve their critical interactions with other species. The high number of variants of QS synthases identified in CPR and DPANN genomes also offers a promising reservoir for the discovery of new molecules of communication and suggests that the multiplicity of “dialects” in the microbial world is probably larger than previously appreciated.

## MATERIALS AND METHODS

### Construction of a reference database of sequences and HMMs of QS synthases and receptors.

We carefully mined the literature related to QS to establish a list of QS systems. We notably relied on the Sigmol (32) and the Quorum Peps (61) databases of QS metabolites and QS peptides, respectively. This reference list of experimentally characterized QS proteins is available as a tabular file (see sheet 1 of Table S1 in the supplemental material) and summarizes for each protein its function and its NCBI or Uniprot identifier (ID) as well as the QS signal with which it is associated. When available, HMMs of the domains that are specific for QS proteins were retrieved from Pfam (62) and Cath-Gene3d (63) and were further assembled in a library using the hmmpress tool from HMMER suite version 3.2.1 (64) (Table S1, sheet 2). We also constructed our own HMM profiles from the query protein families that comprised more than 5 validated QS proteins (e.g., LuxS, LuxI, etc.) and mined the literature of structural studies related to well-studied QS families in order to construct HMM profiles of domains that are specific to QS proteins (AHL-binding domain of LuxR, periplasmic domain of QseC, etc.) (Table S1, sheet 2). Each HMM profile was built as follows: the multiple-sequence alignment of either full sequences or domains of QS proteins was built using MUSCLE version 3.8.31 (65) and was given as input to the hmmbuild tool (the HMMER suite version 3.2.1; 64). All resulting HMMs were compiled together in a library using hmmpress.

### Retrieval of the protein sequences of the CPR and DPANN genomes.

All the protein sequences of the CPR and DPANN genomes were downloaded from the NCBI assembly database as of 17 December 2019 (66). At the time of writing, these CPR genomes/proteomes were able to be retrieved from the lineages corresponding to the following taxonomic IDs: txid74243, txid95818, txid221235, txid363464, txid422282, txid1618330, txid1618338, txid1618339, txid1618340, txid1619053, txid1794810, txid1794811, and txid1817799. The DPANN genomes/proteomes can be retrieved from the lineages corresponding to the following taxonomic IDs: txid1462430, txid1783276, and txid1803511. All the taxonomic information on these genomes is given in Table S6.

10.1128/mSystems.00414-20.9 TABLE S6 CPR and DPANN genomes considered in this study. Download Table S6, XLSX file, 0.2 MB. Copyright © 2020 Bernard et al. 2020 Bernard et al. This content is distributed under the terms of the Creative Commons Attribution 4.0 International license.

### Detection of homologs of reference QS proteins in CPR and DPANN.

The homologs of reference, experimentally validated QS proteins in CPR and DPANN, were identified with BLASTp version 2.2.31+ (67), using as queries all the protein sequences composing the reference QS protein families (Table S1) and as targets all the retrieved protein sequences corresponding to the publicly available CPR and DPANN genomes. Homology was classically assessed according to the following thresholds: sequence identity, ≥25%; E value, <1e−5; mutual alignment coverage, ≥75% (30, 31). Homologs identified with an E value of <1e−20 were assigned a specific “significance” label in the corresponding heat map (Fig. 2). Homologs found at the first BLAST iteration were used as queries in a second BLAST search, using the same thresholds but imposing the restriction that the remote homologs must exhibit ≥75% mutual coverage with the initial, reference QS proteins. Independently, the HMMs of QS proteins were searched against the library of CPR and DPANN protein sequences using hmmsearch from the HMMER suite (64). Only the hits yielding an E value of <1e−4 were retained, representing a threshold which is 100 times more stringent than the default inclusion threshold. Homologs identified by both BLAST and hmmsearch were highlighted in the corresponding heat map (Fig. 2).

### Detection of homologs of the CPR and DPANN variants of QS proteins in the complete genomes of *Bacteria* and Archaea.

The protein sequences corresponding to all available complete genomes of *Archaea* and representative *Bacteria* were retrieved from the NCBI assembly database 16 July 2019. The best homolog of each CPR/DPANN variant of a QS protein was identified using the same method as described above, albeit with no second BLAST iteration. The comprehensive list of all query QS proteins and the CPR/DPANN variants and their respective best matches in reference prokaryotes is given in Table S2.

### Multiple-sequence alignment (MSA) of each family of homologs.

All the homologs identified by a query family of QS proteins were aligned together to further compute their associated *dN*/*dS* ratio. For visualization purposes, the MSAs displayed in Fig. 3 were built only from the best homolog (lowest E value) identified in each CPR or DPANN phylum. Jalview was then used to display the conservation level of the important residues (68). In both cases, the MSAs were built using Muscle version 3.2.1 with the option “-maxiters 50” (65).

### *dN*/*dS* ratio of each family of homologs.

The MSA of each family of homologs identified in the CPR and DPANN lineages was trimmed by the use of trimAl version 1.4.rev22 with the options “-gt 0.8” and “colnumbering” to retain only the positions with less than 20% of gaps (69). The NCBI IDs of the protein sequences in the alignment were used to fetch the corresponding coding DNA sequences (CDS) from the NCBI, via the “efetch -db protein -format fasta_cds_na -id <protein_id>” E-utilities command line. We generated the MSA of CDS based on the protein following MSA: gaps were substituted by the “—” characters and amino acids by their corresponding codons. Accordingly, the stop codons were not represented in the MSA of CDS. Then, we introduced a slight modification in the source code of the SNAP perl script, a tool to compute synonymous and nonsynonymous values for a MSA of CDS (70). Namely, in the dictionary which associates an amino acid letter to a cognate codon, we substituted the dummy “Z” character associated with the opal “UGA” stop codon by a “G,” to account for the alternative genetic code of “*Candidatus* Gracilibacteria” and “*Candidatus* Absconditabacteria,” in which the opal codon encodes a glycine (NCBI:transl_table = 25). Of note, the other DPANN and CPR phyla were not reported to rely on an alternative genetic code. The *dN*/*dS* ratio of each family of homologs was then given as output by the thus-modified SNAP tool as the average of the *dN*/*dS* ratios computed for each possible pairwise comparison of sequences in the alignments.

### Distribution of the homologs in the CPR and DPANN phyla.

The multi-informative heat map (Fig. 2) was generated using the ComplexHeatmap R package version 1.99.5 (71). Two matrices were superimposed; the first one corresponds to the number of homologs identified in each phylum, normalized by the number of genomes in the phylum (grayscale background), whereas the second corresponds to the percentage of identity that is representative of the best match (lowest E value) between the proteins of a query family and their homologs identified in a phylum (colorscale foreground).

### Secrete and sense tripartite network.

The protein sequences of each family of homologs were processed in a BLAST All versus All search (serving as both queries and targets) (67). Only the pairs of proteins showing a percentage of identity of ≥90% over 75% mutual coverage were retained. Based on these pairs of highly similar proteins, a network was built to identify the nodes (proteins) that are connected to each other, thereby forming connected components (a group of highly similar proteins). The subfamilies were defined according to the distinct connected components identified in the network (Table S5, sheet 1). Each subfamily was further assigned a “divergence” indicator, defined as the average of the percentages of sequence identity between the sequences of the CPR/DPANN subfamilies and their respective best match in the reference prokaryotes. Subsequently, an “Edge” table was constructed to reference the membership of each genome in the different subfamilies (Table S5, sheet 2). A “Type” table was also constructed to assign a level to each node of the “Edge” table as follows: level 1 for the subfamilies of putative QS synthases, level 2 for the genomes, and level 3 for the subfamilies of putative QS receptors (Table S5, sheet 3). All the edges were considered in the DPANN tripartite network whereas only the connected components of size >3 were retained in the CPR network. These two tripartite networks were plotted with the igraph R package version 1.2.4 (72), using the Sugiyama algorithm to display the three levels of nodes in layers and to minimize the entanglement of the edges in the two-dimensional (2D) projection of each graph (73).

### Data availability.

All the CPR/DPANN homologs of QS proteins, as well as the taxonomic information on the genomes discussed here, are given in the supplemental material.
