# Supplementary figures and images for: Rich Repertoire of Quorum Sensing Protein Coding Sequences in CPR and DPANN Associated with Interspecies and Interkingdom Communication
Source: mSystems. 2020 Oct 13;5(5):e00414-20. doi: 10.1128/mSystems.00414-20 (PMC7567580; doi:10.1128/mSystems.00414-20)

Figure S1

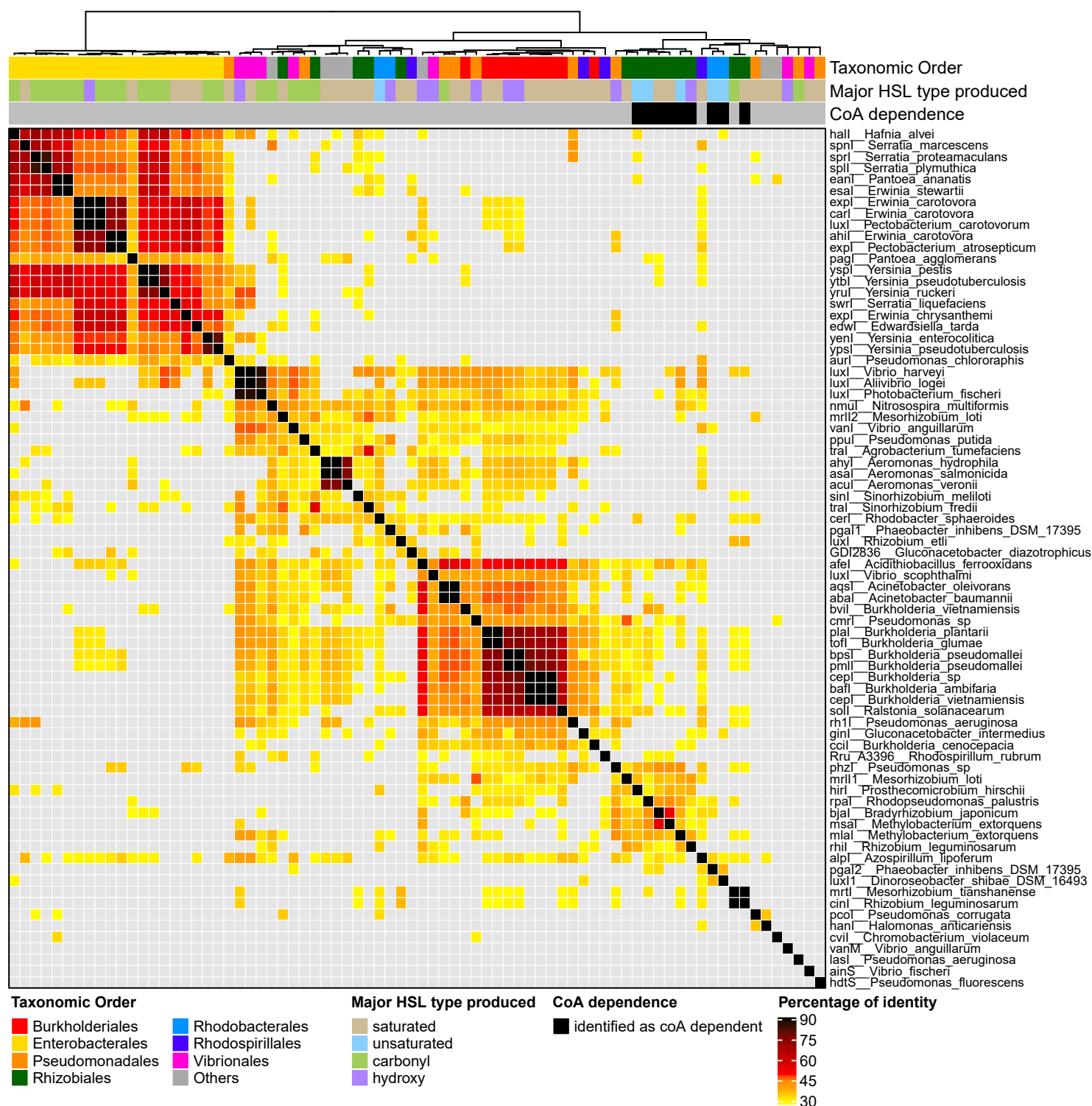

Supplement: FIG S1 [file mSystems.00414-20-sf001.pdf]

*Figure S2*

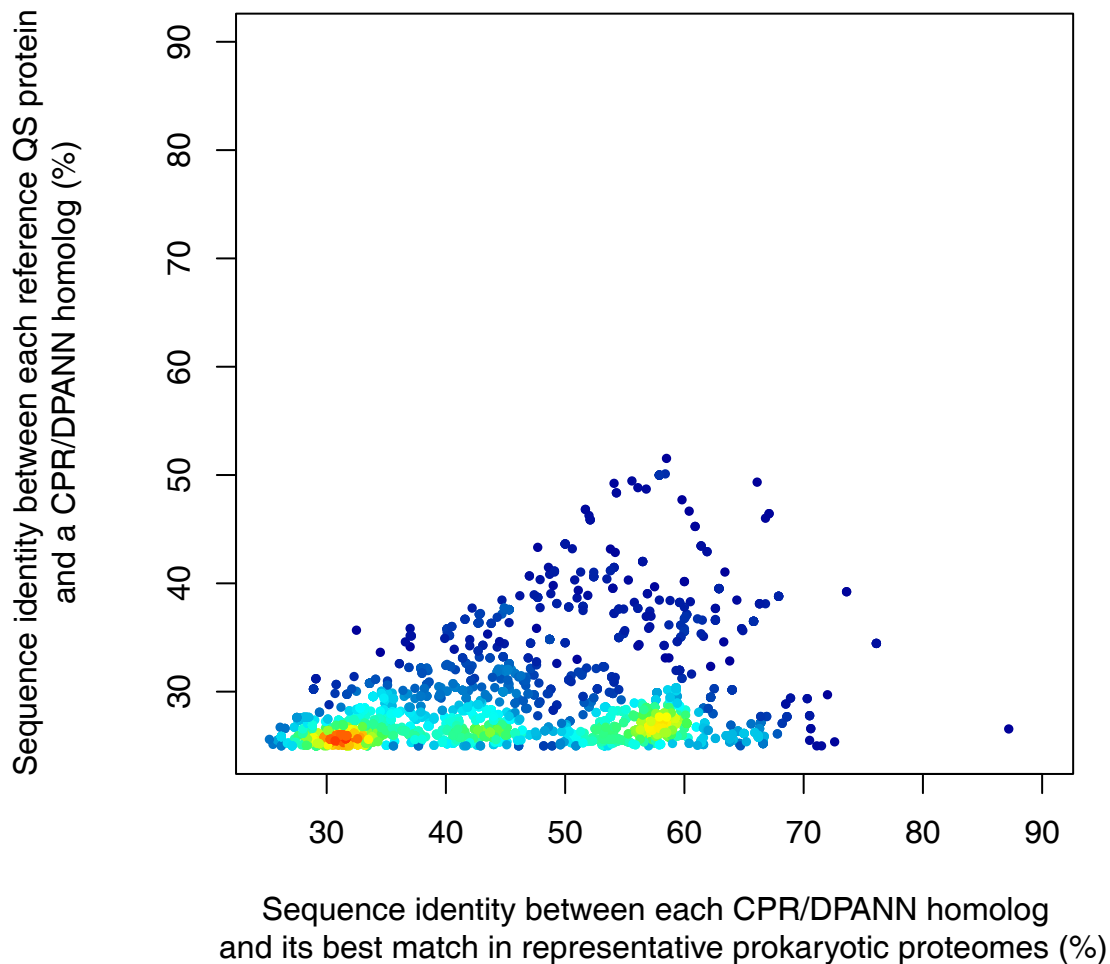

Supplement: FIG S2 [file mSystems.00414-20-sf002.pdf]

Figure S3

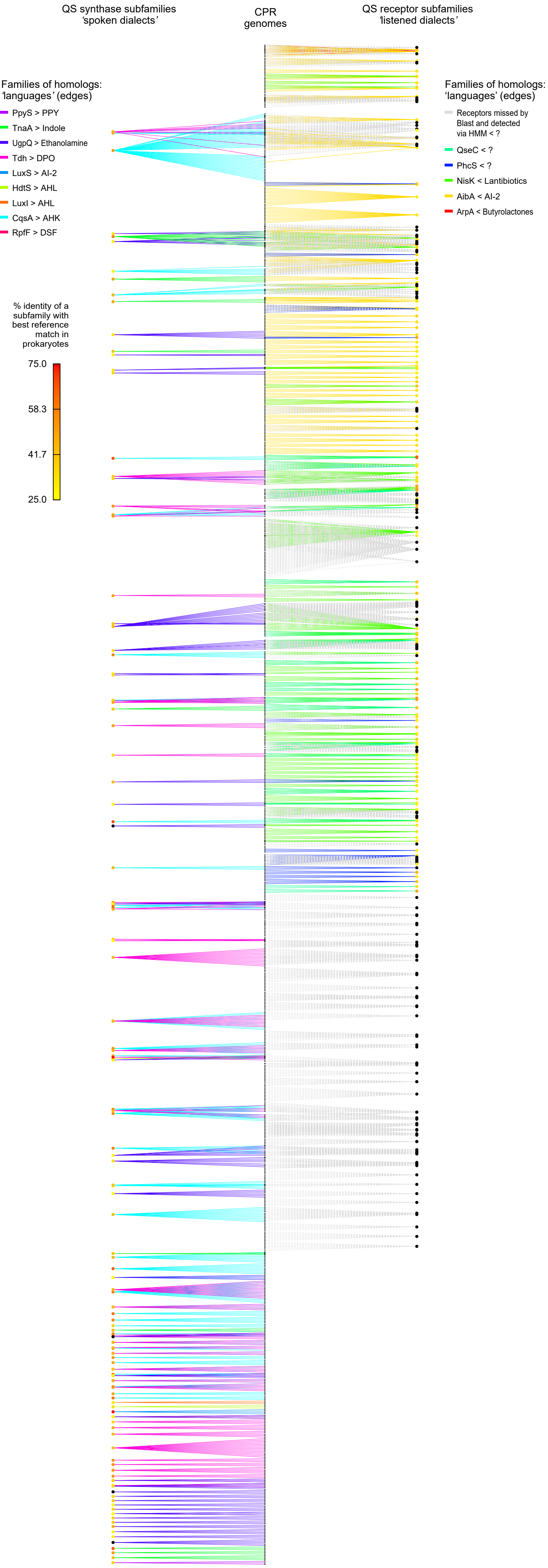

Supplement: FIG S3 [file mSystems.00414-20-sf003.pdf]
